# Proteomics Sample Preparation, Preservation, and Fractionation

**DOI:** 10.1155/2012/701230

**Published:** 2012-12-30

**Authors:** Gary B. Smejkal

**Affiliations:** ^1^Covaris Inc., 14 Gill Street, Suite H, Woburn, MA 01801, USA; ^2^Hubbard Center for Genome Studies, University of New Hampshire, Durham, NH, USA

In eukaryotic cells, protein synthesis occurs at the rate of 6–9 amino acid residues per second. With a median length of 360 amino acids, the synthesis of an “average” protein takes about a minute to complete [1]. At this rate, the synthesis of a single molecule of the muscle protein titin, being over 34,000 residues in length, requires over two hours to complete [2]. While this seems slow as biological processes go, the cellular requirement for protein synthesis is satisfied by the huge numbers of ribosomes, which can comprise 30% of a cell's total mass [3]. Human HeLa cells, for example, can contain over nine million ribosomes [4]. Extrapolated from the finding that as many as 80% of the ribosomes can be actively synthesizing protein in metabolically active cells [5], a single cell could theoretically generate 120,000 protein molecules per second.

In its November 2012 release statistics, UniProt/trEMBL reported 28,395,832 sequence entries in its protein database [6]. At the rate of six amino acids per second, a single eukaryotic ribosome working non-stop would require over 48 years to translate the entire database. However, there is protein evidence for only 0.05% and RNA transcript evidence for only 2.21% of the total entries [6]. With fewer than 112,000 sequence entries, *Homo sapiens* comprises only 0.04% of the total sequence entries. It would seem that human proteomics is not in its infancy, it is embryonic.

The number of human proteins is expected to reach into the millions. Immunoglobulins alone are encoded from 70 genes for which there are 320 possible light chain combinations and 10,530 possible heavy chain combinations resulting in 3,369,600 possible quaternary structures [7]. In even the simplest of organisms, the broad concentration of protein expression frequently spanning over nine orders of magnitude compounds the complexity of the proteomic amalgam. An undeterminable number of possible post-translational modifications that produce multiple isoforms of many proteins add another layer of complexity. For instance, there are 3778 distinct genes encoding plasma proteins of which at least 51% of these genes encode more than one protein isoform [8]. Hence, neither genomics nor transcriptomics can reliably predict the protein constituents of cells, tissues, or biological fluids. 

The search for biologically important proteins of low abundance is impeded by the enormous range of protein concentrations, as exemplified in human plasma where the mass of albumin is nearly ten billion times greater than that of important signaling proteins such as the interleukins [9, 10]. The diversity of proteins, ranging from very soluble proteins in biological fluids to extremely hydrophobic ones that exist either embedded in lipid membranes or as insoluble aggregates, suggests that the total protein constituency of cells may not be isolated without bias towards or against some protein subpopulations. On the other hand, the complexity of proteomes might be selectively decreased by exploiting the bias toward specific protein subpopulations.

Lessons learned from early computer programmers who coined the phrase “Garbage in, garbage out”, downstream proteomics analyses are only as reliable as the upstream sample preparation. 

“We now have the technical ability to get the wrong answers with unprecedented speed,” commented Carolyn Compton, former Director of the National Cancer Institute, Office of Biorepositories and Biospecimen Research. “If we put the wrong stuff into the front end of our analytical pipeline, we'll pollute the scientific literature with incorrect data that will take us a long time to sort out.” [11].

This special issue dedicates to the challenges of sample preparation in the proteomics era. This issue convenes several leaders in the field of proteomics as guest editors, authors, and reviewers whose contributions have culminated in making this a most substantive work. The articles within this Special Issue are timely and will be of particular interest to the field.
